# Productively Infected Murine Kaposi's Sarcoma-Like Tumors Define New Animal Models for Studying and Targeting KSHV Oncogenesis and Replication

**DOI:** 10.1371/journal.pone.0087324

**Published:** 2014-01-28

**Authors:** Brittany M. Ashlock, Qi Ma, Biju Issac, Enrique A. Mesri

**Affiliations:** 1 The Miami Center for AIDS Research, Sylvester Comprehensive Cancer Center, University of Miami Miller School of Medicine, Miami, Florida, United States of America; 2 Department of Microbiology and Immunology, Sylvester Comprehensive Cancer Center, University of Miami Miller School of Medicine, Miami, Florida, United States of America; 3 Viral Oncology Program, Sylvester Comprehensive Cancer Center, University of Miami Miller School of Medicine, Miami, Florida, United States of America; 4 Division of Bioinformatics, Biostatistics and Bioinformatics Core, Sylvester Comprehensive Cancer Center, University of Miami Miller School of Medicine, Miami, Florida, United States of America; Duke University Medical Center, United States of America

## Abstract

Kaposi's sarcoma (KS) is an AIDS-defining cancer caused by the KS-associated herpesvirus (KSHV). KS tumors are composed of KSHV-infected spindle cells of vascular origin with aberrant neovascularization and erythrocyte extravasation. KSHV genes expressed during both latent and lytic replicative cycles play important roles in viral oncogenesis. Animal models able to recapitulate both viral and host biological characteristics of KS are needed to elucidate oncogenic mechanisms, for developing targeted therapies, and to trace cellular components of KS ontogeny. Herein, we describe two new murine models of Kaposi's sarcoma. We found that murine bone marrow-derived cells, whether established in culture or isolated from fresh murine bone marrow, were infectable with rKSHV.219, formed KS-like tumors in immunocompromised mice and produced mature herpesvirus-like virions *in vivo*. Further, we show *in vivo* that the histone deacetylase (HDAC) inhibitor suberoylanilide hydroxamic acid (SAHA/Vorinostat) enhanced viral lytic reactivation. We propose that these novel models are ideal for studying both viral and host contributions to KSHV-induced oncogenesis as well as for testing virally-targeted antitumor strategies for the treatment of Kaposi's sarcoma. Furthermore, our isolation of bone marrow-derived cell populations containing a cell type that, when infected with KSHV, renders a tumorigenic KS-like spindle cell, should facilitate systematic identification of KS progenitor cells.

## Introduction

Kaposi's sarcoma (KS) was first described by Moritz Kaposi in 1872 [1], [2]. Over a century and a half later, a substantial increase in patients presenting with KS in New York and Los Angeles heralded the beginning of the AIDS pandemic and led to the discovery of KS-associated herpesvirus (KSHV/HHV-8) as the etiologic agent of the disease [3], [4]. KS is one of three known AIDS-associated malignancies caused by KSHV, with primary effusion lymphoma (PEL) and multicentric Castleman's disease (MCD) being the other two [5], [6]. KS is not only an AIDS-defining cancer; it is also the most common AIDS-associated cancer.

KS is classified into 4 clinical forms: classical, endemic, iatrogenic and epidemic AIDS-associated that are histologically indistinguishable and are characterized into: patch, plaque and nodular, with the acceptance that these morphologies represent a continuum and not necessarily distinct entities [7], [8]. Histologically, the tumor is composed of inflammatory infiltrates, KSHV-infected cells of spindle morphology (e.g. the pathognomonic spindle cell), and aberrant angiogenesis with extravasated red blood cells (RBC) in slit-like spaces. The origin of the spindle cell continues to be an enigma in KS research [9]–[11]. Although they express vascular and lymphatic endothelial markers, they also express markers that belong to hematopoietic and endothelial progenitor cells (EPCs). Therefore, they are believed to have either a progenitor origin or are originated by KSHV-induced transdifferentiation of a committed endothelial lineage cell [9], [10], [12], [13]. Lytic replication not only ensures the production and spread of virions within and between hosts [12], it allows for the expression of pathogenic lytic genes, some of which have proposed roles in the paracrine neoplasia thought to drive the tumor [14]–[16].

Current treatment for KS is largely reliant upon either HAART therapy or systemic chemotherapeutic agents, both of which, that can have significant side effect profiles [17]–[19]. Although most occurrences of AIDS-KS initially respond to HAART, HAART refractory tumors are treated with systemically cytotoxic chemotherapies. Regardless of treatment modality, disease recurrence is generally within a year and complete remission is rarely seen [20], [21].

Ideally, the KSHV- infected cells are perfect substrates for rationally designed therapies, as the virus contributes numerous non-host molecular targets and processes [22]. A limitation to the use of antivirals targeting the KSHV-lytic replication, is that the majority of the cells in a KS lesion harbor latent virus, effectively avoiding the immune system [23]. KSHV latency is sustained by the latency associated nuclear antigen (LANA) which allows for KSHV genome persistence and immune evasion [24], [25]. A way of enhancing the efficaciousness of antiviral therapies against latent viruses is to induce the virus into lytic replication [26], [27]. Unfortunately, studies of lytic replication are reliant upon chemical induction *in vitro*, are limited because as they do not recapitulate the temporally ordered cascade of replicative events and lack the *in vivo* host environment in which KSHV has evolved [28]–[31]. Indeed, antivirals that have proven efficacious generally target the KSHV DNA polymerase or viral thymidine kinase during the lytic portion of the replicative cycle [32]–[34]. Our own recent study showed that potent induction of the lytic cycle with Vorinostat (suberoylanilide hydroxamic acid/SAHA) and Bortezomib (Btz) led to massive apoptosis of primary effusion cells (PEL) *in vitro* and *in vivo*, without an increase in viral load *in vivo* and with a concurrent increase in the life span of the murine host [35]. Thus, Kaposi sarcoma models that can reliably recapitulate KSHV lytic replication, particularly reactivation and virion production in vivo are needed to better understand KSHV-induced pathogenesis along with the testing of new targeted antiviral strategies for Kaposi sarcoma.

Current animal models to study KS pathogenesis have led to major advances in the field, but each has limitations in its ability to fully recapitulate KSHV-induced viral oncogenesis [36]–[40]. Our lab developed an *in vitro* and an *in vivo* model, termed mECK36. Here, murine bone marrow-derived endothelial lineage cells (mEC) were transfected with BAC36, a bacterial artificial chromosome harboring the full KSHV genome, leading to the creation of the mECK36 cell population [28], [41]. When the mECK36 cells were subcutaneously injected into immunodeficient mice, KS-like tumors developed within weeks. Although mECK36 sarcomas were composed of latently and lytically infected spindle cells, a major limitation of the mECK36 model is the inability to complete a full replicative cycle as determined by lack of virion production [28]. Nevertheless, this work pointed to the existence of a subpopulation of bone marrow resident cells in which KSHV infection would have an oncogenic outcome.

Herein, we sought to identify whether such a mECK36 like cell type could be created by *de novo* infection leading to productively infected tumors using a two-step approach. First, we evaluated whether a recombinant infectious KSHV, rKSHV.219, could replace BAC36 in exactly the same cellular environment to yield tumorigenic cells and whether, in these cells, rKSHV.219 could complete a full replicative cycle. Second, we tested whether we could reproduce this by direct infection of marrow resident cells isolated in the same manner as the mECs from our original model of KSHV-induced tumorigenesis.

By replacing the BAC36 construct in our mECK36 model with rKSHV.219, we generated a tumorigenic cell line that when injected in immunodeficient mouse forms KS-like tumors displaying herpesvirus-like particle (HVLP) as detected by electron microscopy. Next, we found that *de novo* rKSHV.219 infection of primary bone marrow-derived cells lead to cellular immortalization in culture and transformation *in vivo* with robust, KS-like tumor development in immunocompromised mice, opening up the possibility of using genetically modified mice to elucidate host contributions to KS pathogenesis. Finally, using a clinically relevant histone deacetylase (HDAC) inhibitor, we show that, KSHV reactivation can be enhanced *in vivo*, a result desirable to test many emerging antiviral therapeutic approaches.

## Results

### The loss of BAC36 from the tumorigenic mECK36 cells results in the non-tumoigenic population, mECK^null^

**Figure 1:**
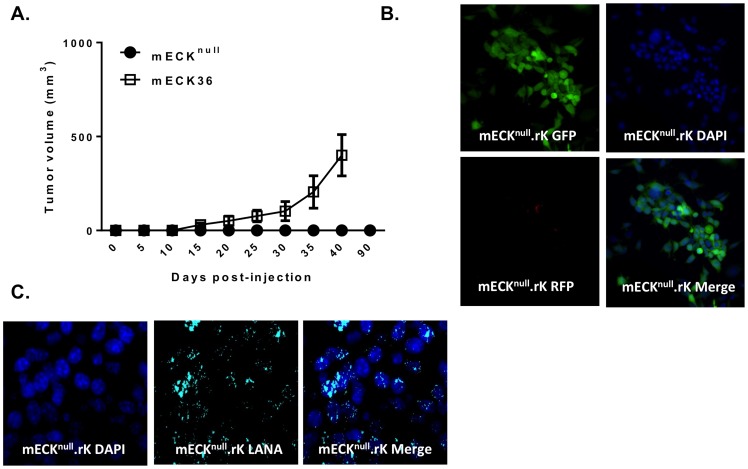
Replacing BACK36 in mECK36 cells for rKSHV.219 generates the tumorigenic cells, mECK^null^.rK. (A) Graph depicting mECK36 tumor kinetics in athymic nu/nu mice. 3×10^6^ mECK36 cells were subcutaneously injected into the hind flanks of 3 athymic nu/nu mice. Concurrently, 3×10^6^ mECK^null^ cells were subcutaneously injected into another group of 10 athymic nu/nu mice. Within 4–6 weeks solid mECK36 tumors were palpable and growth was monitored by caliper measurement (open squares). Error bars represent the standard deviation between 3 different tumors. mECK^null^ cells did not form tumors (black dots). (B) Fluorescence microscopy of mECK^null^.rK. *In vitro*, mECK^null^.rK express GFP constitutively, indicating rKSHV.219 infection, and maintain tight latency as determined by the absence of RFP expression. (C) Cell cultures of mECK^null^.rK were prepared for immunofluorescence for the KSHV LANA which exhibited the classic punctate nuclear pattern of the protein.

**Figure 2:**
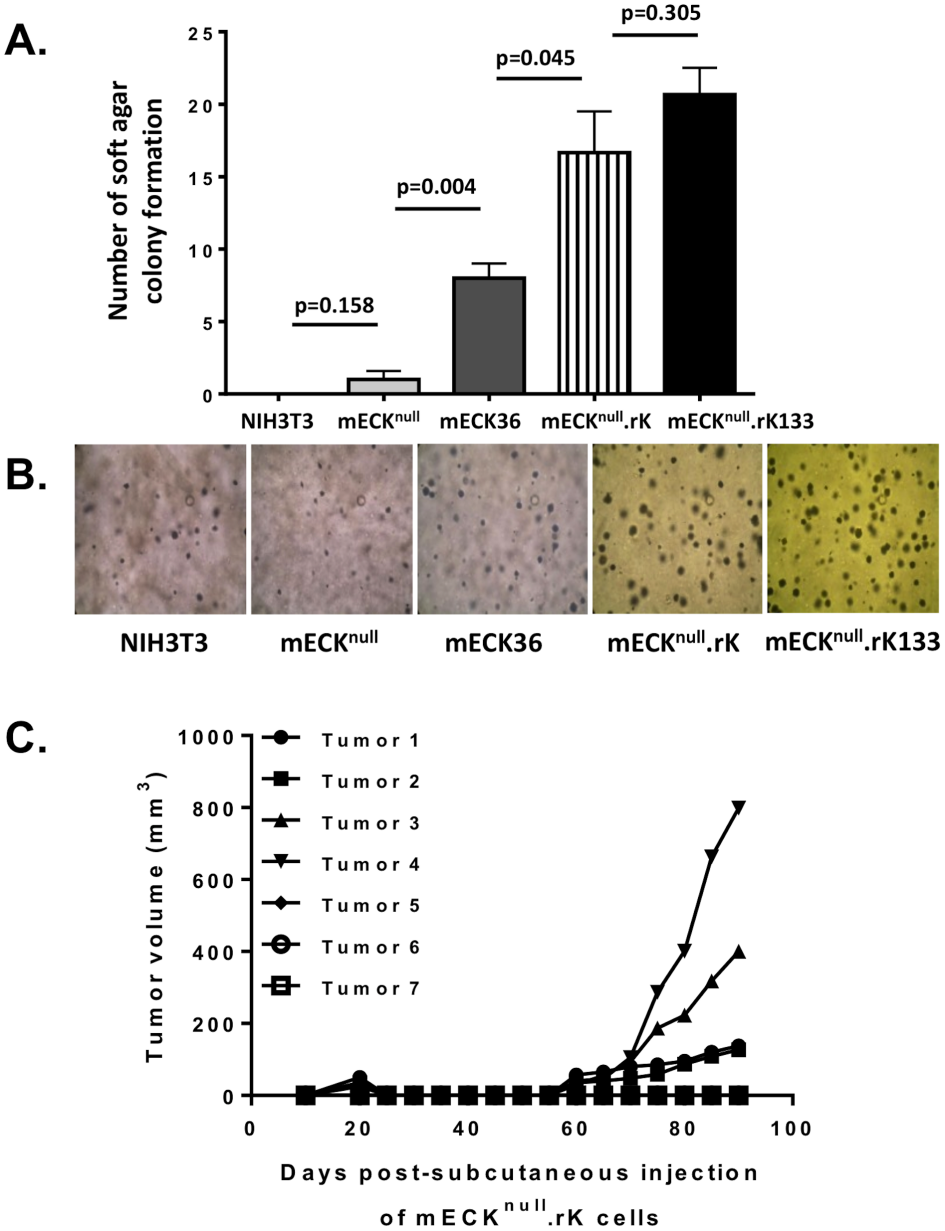
mECK^null^.rK exhibit variable tumorigenicity. (A) A graph depicting number of colonies formed in soft agar at 4 weeks from 5000 single cells originally plated. Error bar represents standard deviation of number of colonies counted in 10 separate fields imaged at 10×. NIH 3T3 cells and mECK^null^ cells do not form appreciable colonies while mECK^null^.rK and mECK^null^. rK133 cells exhibit robust colony formation. Error bars represent the SD between triplicate wells. (B) Representative image at 10× magnification of colonies in soft agar taken 4 weeks after plating single cell suspensions of NIH3T3, mECK^null^, mECK36, mECK^null^.rK and mECK^null^. rK133. (C) Graph depicting mECK^null^.rK tumor kinetics in athymic nu/nu mice. 3×10^6^ mECK^null^.rK cells were subcutaneously injected into the hind flanks of 7 athymic nu/nu mice. 8 weeks later solid tumors were palpable in 4 of the 7 mice. Growth was monitored by caliper measurement.

We overcame the limitations of our mECK36 cells, by replacing the transfected BAC36 construct with an infection of rKSHV.219, a fully replication competent recombinant KSHV [42]. This was done sequentially. First, we cultured the mECK36 cells without hygromycin selection, allowing for the loss of the episomal BAC36. The loss of the BAC36 was confirmed by flow cytometry for GFP showing gradual loss of GFP until the population was totally GFP negative [28] and by a negative PCR for LANA (not shown). This resulted in the non-tumorigenic KSHV-null mECK36 cell line, mECK^null^, first described in Mutlu *et al.* and reproduced in these sets of experiments [28]. Two major pieces of evidence indicated that the mECK^null^ were not transformed: (1) there was no tumor formation up to three months after subcutaneous injection of 3 million cells into nude mice (0/10), while an equivalent number of mECK36 cells form subcutaneous tumors that are palpable after just two weeks (Figure 1A), (2) the mECK^null^ cells lost the ability to form colonies in a soft agar assay while the mECK36 cells remained colony forming in soft agar (Figure 2A, B) and, (3) the cells remained contact inhibited *in vitro* (not shown). These assays confirmed a concurrent loss of tumorigenicity with loss of the BAC36 KSHV genome and indicated that mECK^null^ cells remained an appropriate substrate for studies of KSHV-induced tumorigenicity.

### Replacing BAC36 in mECK36 cells for rKSHV.219 preserves KSHV-dependent tumorigenesis

mECK^null^ cells were infected with rKSHV.219 at a multiplicity of infection (MOI) of 1 to generate the cells we termed mECK^null^.rK. rKSHV.219 is a construct created from wild type KSHV isolated from JSC-1 cells and modified to constitutively express GFP driven by an EF-1α promoter, along with the *pac* gene allowing for puromycin selection. The recombinant virus also contains RFP driven by the KSHV replication and transcriptional activator (RTA)-dependent lytic polyadenylated nuclear (PAN) RNA promoter, allowing for the identification of cells undergoing early lytic replication [42]. The mECK^null^.rK were cultured for two weeks under puromycin selection resulting in a puromycin resistant population of KSHV infected mECK^null^.rK cells that were >98% GFP positive (Figure 1B), with very few cells undergoing lytic replication, as determined by RFP expression (Figure 1B). Further, the mECK^null^.rK expressed LANA in the classic punctate nuclear pattern characteristic of KSHV episomal infections (Figure 1C).

**Figure 3:**
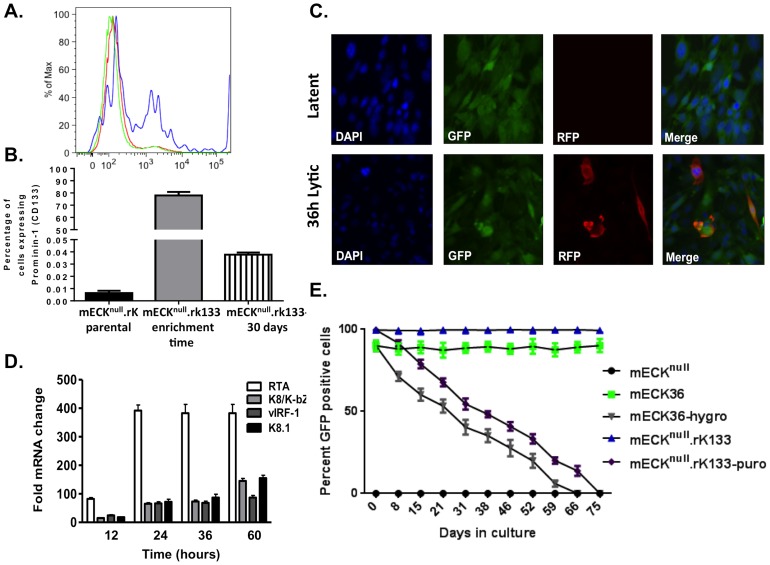
CD133/Prominin-1 enrichment of the mECKnull.rK results in a lytically-inducible population that harbors KSHV as an episome. (A) mECK^null^.rK were harvested from cell culture and CD133-expressing cells were positively selected using immunomagnetic beads. The histogram shows the parental population (green), the CD133 enriched (mECK^null^.rK133) (blue) and the CD133-depleted populations (red) were analyzed by flow cytometry to confirm CD133 enrichment. A representative histogram is shown. (B) Graph depicting the percentage of CD133 expression in the mECK^null^.rK, prior to CD133-enrichment, the percentage of CD133 in the enriched population of mECK^null^.rK133 immediately post-enrichment, and the mECK^null^.rK133 one month after CD133 enrichment. Error bars represent standard deviation between triplicate wells. (C) *In vitro*, mECK^null^.rK133 express GFP, indicating rKSHV.219 infection, and maintain tight latency as determined by the absence of RFP expression (top panels). When treated with TSA lytic replication is induced as determined by the expression of RFP (bottom panels). (D) RFP induction after lytic reactivation is concurrent with the upregulation of KSHV lytic gene expression as determined by qRT-PCR for RTA, K8, vIRF-1 and K8.1. Error bars represent the SD of duplicate wells. Data are representative of three independent experiments. (E) mECK^null^.rK133 cells are episomally-infected with KSHV. mECK^null^.rK133 cells were serially passaged in the absence of hygromycin and GFP was measured by flow cytometry. While almost 100% of cells are GFP positive at day 0, by day 70, they are 100% GFP negative, suggesting that the virus exists as an episome in the murine cells.

mECK^null^.rK were examined for anchorage-independent cell growth in a soft agar assay to determine whether they displayed changes in their ability to form colonies upon rKSHV.219 infection. Like the mECK36, the mECK^null^.rK formed significantly more colonies at 6 weeks than the control and the mECK^null^ cells (Figure 2A, B) suggesting that the presence of the viral genome conferred a transformed phenotype. Subcutaneous injection of mECK^null^.rK cells into nude mice resulted in the mECK^null^.rK forming tumors only 60% of the time (4 mice, N = 7) by 8 weeks and with broadly variable growth rates (Figure 2C). As mECK36 originated from endothelial-lineage cells, in an attempt to obtain a population of cells that were more uniformly tumorigenic, mECK^null^.rK were enriched for murine CD133/Prominin, an EPC marker found to be upregulated by KSHV infection [43]–[45] using positive selection with immunomagnetic beads (Figure 3A, B). These cells were further expanded under puromycin selection for tumorigenesis studies. We termed this expanded population of CD133-enriched cells, mECK^null^.rK133 cells. FACS analysis showed that even upon expansion in culture for 30 days, mECK^null^.rK133 cells continued to show a 7-fold enrichment over the mECK^null^.rK (Figure 3B). Colony formation in soft agar was confirmed as with the previous cells (Figure 2A, B). A general characteristic of KSHV-infected endothelial lineage cells is that, *in vitro*, the virus is undergoing lytic replication in just a minority of cells at any one time [46]. This is also the case with mECK^null^.rK133. We observed tight maintenance of latency *in vitro*, as determined by an absence of RFP expression (Figure 3C, top panels). To evaluate the ability of the mECK^null^.rK133 to support lytic replication, they were induced with the histone deacetylase (HDAC) inhibitor, Trichostatin A (TSA), for 12, 24, 36 and 60 hours. When undergoing lytic replication the rKSHV.219 produces RFP; about 5% of cells underwent lytic replication as determined by RFP expression at 36h (Figure 3C, bottom panels). The induction of RFP expression correlated with a sustained increase in RTA and other lytic KSHV transcripts (Figure 3D).

To verify that, as suggested by the punctuated LANA IFA staining, the rKSHV.219 infection was episomal in mECK^null^.rK133 cells, they were cultured in the absence of puromycin and GFP expression was followed by flow cytometry. By week 10 the population was KSHV negative as determined by flow cytometry for GFP (Figure 3E) and PCR for LANA (not shown). The parental mECK36 and descendent mECK^null^.rK cells also harbored the KSHV genome as an episome, as these cells also lost GFP expression in a time and passage dependent manner (Figure 3E) along with the loss of LANA (not shown) with passage in non-selective medium.

### mECK^null^.rK133 form productively infected KS-like tumors in immunocompromised mice

**Figure 4:**
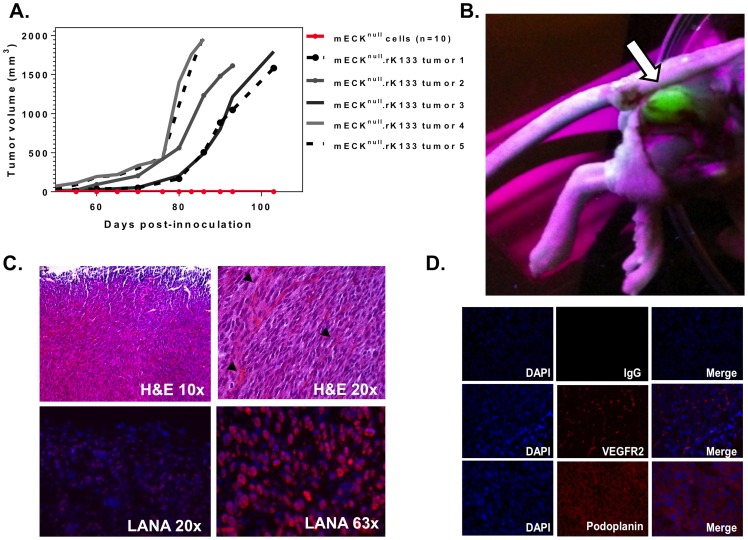
mECK^null^. rK133 cells consistently form KS-like tumors in immunocompromised mice. (A) Graph depicting tumor kinetics in athymic nu/nu mice. 3×10^6^ mECK^null^. rK133 cells were subcutaneously injected into the hind flanks of 5 athymic nu/nu mice. Concurrently, 3×10^6^ mECK^null^ cells were subcutaneously injected into another group of 10 athymic nu/nu mice. Within 4–6 weeks solid mECK^null^. rK133 tumors were palpable and growth was monitored by caliper measurements. mECK^null^ cells did not form tumors (red line, x-axis). (B) Dissection site showing a GFP+ tumor indicating the presence of rKSHV.219. The subcutaneous tumor was visualized under UV. (C) Tumor morphology was analyzed by H&E staining of paraffin embedded sections. Pathologically, they are composed of spindle cells arranged in bundles with RBCs in slit-like vasculature (top panels, black arrows). Frozen sections were prepared for immunofluorescence for KSHV LANA which showed that the spindle cells of the tumor express the viral latent nuclear antigen, LANA (bottom panels). (D) Immunofluorescence analysis of angiogenic protein expression *in vivo* reveals that the tumor cells express VEGF-R2 and podoplanin. A representative tumor is shown.

To determine if the mECK^null^.rK133 maintained tumorigenicity, we subcutaneously injected an equivalent number of cells into the hindflanks of nude mice. Although the colony formation assays showed no significant difference between mECK^null^.rK and mECK^null^.rK133, the mECK^null^.rK133 cells displayed a more homogeneous and reliable tumorigenicity. Solid tumors were palpable between four and six weeks post-injection (Figure 4A) with an incidence of 95% (38/40). Upon dissection, vibrantly GFP positive tumors were visualized under ultraviolet light (Figure 4B). Paraffin-embedded sections were stained with hematoxylin and eosin (H&E) and evaluated by a pathologist who confirmed that the tumors were indistinguishable from the vascularized spindle-cell sarcomas formed by mECK36 tumors previously generated by our lab which were thoroughly characterized as KS-like tumors. Examination of the H&E images revealed spindle-shaped cells arranged in bundles with extravasated red blood cells in slit-like spaces (Figure 4C, top panels). Using immunofluorescence for the LANA protein of KSHV, it was determined that the majority of the tumor cells displayed the classic punctate nuclear staining while the overlying dermis was LANA negative (Figure 4C, bottom panels). Immunofluorescence analysis revealed that mECK^null^.rK133 tumor cells expressed markers implicated in KS pathogenesis such as the vascular marker VEGF-R2 (Figure 4D, middle panels) and podoplanin, a lymphatic endothelial KS marker, (Figure 4D, bottom panels) but they did not express the EPC marker, CD133 (data not shown). To further determine that the GFP positive tumor cells were of endothelial origin, we stained freshly isolated tumor cells for CD31, a pan-endothelial marker. We found that 40– 60% of the tumor cells, and 90–95% of the GFP+ cells expressed CD31, further confirming the endothelial nature of the KSHV-infected tumor cells (data not shown).

**Figure 5:**
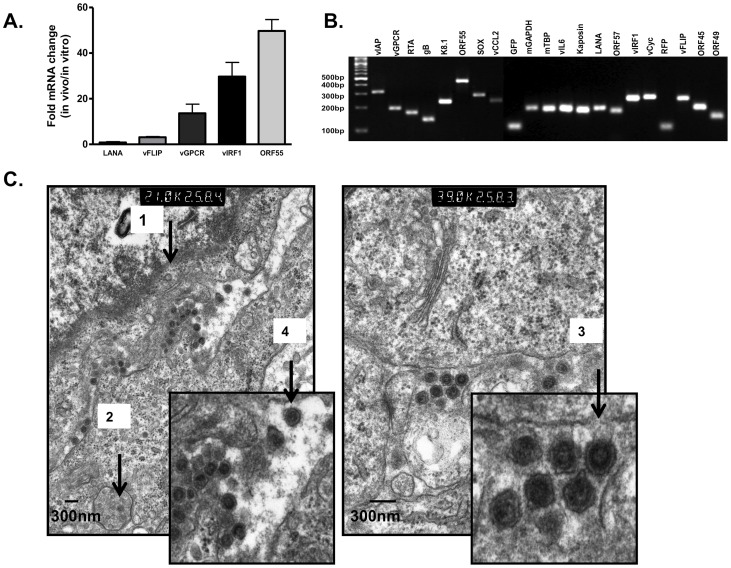
rKSHV.219 lytic replication *in vivo* results in productively infected tumors culminating in the formation of virus-like particles. (A) rKSHV.219 lytic gene expression increases *in vivo* relative to mECK^null^.rK133 cells in culture during tumorigenesis. RNA was isolated from tumors and cells in culture for analysis of rKSHV.219 gene expression by qRT-PCR. A representative comparative analysis is shown; error bars represent the SD of experimental duplicates. (B) RT-PCR analysis of rKSHV.219 transcripts was performed as a preliminary confirmation that the virus was able to express genes representative of the entire viral replicative cycle. RNA was isolated from tumors, reverse transcribed and run on a 3% agarose gel. Gene expression *in vivo* reveals the presence of transcripts that span the entire KSHV genome and replicative potential. Reverse transcriptase negative and non-template controls were run to confirm the absence of contamination. (C) Transmission electron microscopy (TEM) analysis of tumors: Tumors were excised and fixed in gluteraldehyde. TEM revealed the presence of herpesvirus-like particles (100 nm–200 nm) *in vivo*.

To understand the nature of the virus *in vivo* in greater detail, we first analyzed viral lytic gene expression *in vivo* relative to *in vitro* using qRT-PCR. When comparing viral gene expression in mECK^null^.rK133 cells cultured *in vitro* to viral gene expression in mECK^null^.rK133 tumors, we found that the virus was considerably more lytic *in vivo* than in culture as determined by a transcriptional increase of 18-fold, 30-fold and 50-fold in the lytic genes: viral G-protein coupled receptor (vGPCR), viral interferon regulatory factor- 1 (vIRF1) and ORF55, respectively (Figure 5A).

To determine the breadth of KSHV gene expression, specifically, the potential for the full lytic replicative cycle gene set to be transcribed, in mECK^null^.rK133 tumors, 19 viral transcripts spanning the entire viral replicative cycle and genome, were reverse transcribed by RT-PCR and visualized on an agarose gel (Figure 5B). Tumors were next analyzed for rKSHV.219 production by transmission electron microscopy (TEM). Intriguingly, TEM imaging revealed herpesvirus-like particles (HVLPs) between 100–200 nm in size (Figure 5C). We identified intracellular and extracellular HVLPs at varying stages of maturation, including empty capsids (Figure 5C, black arrow 1), HVLPs inside intracellular vesicles (Figure 5C, black arrow 2), some budding from cellular membranes (Figure 5C, black arrow 3) and more mature-appearing particles with glycoprotein spikes (Figure 5C, black arrow 4).

**Figure 6:**
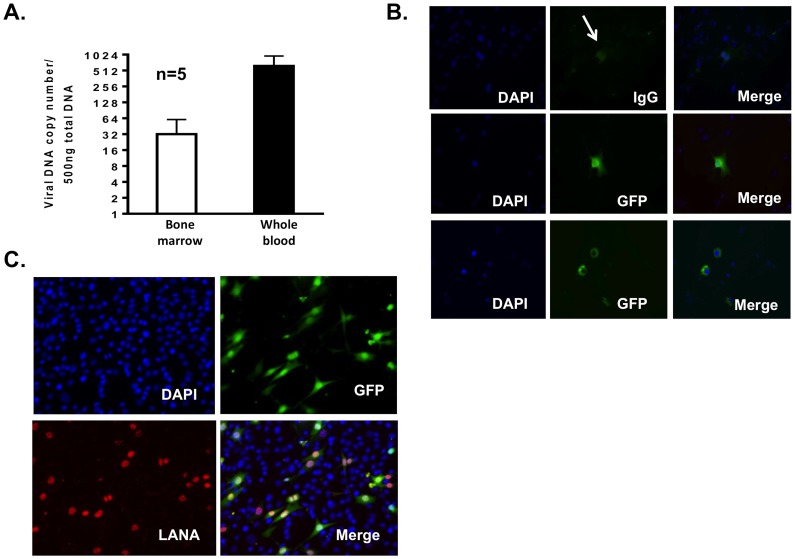
rKSHV.219 infected cells and viral DNA can be detected throughout the murine host. (A) Graph depicts viral DNA copy number per 500 ng total DNA in 10×10^6^ cells purified from bone marrow and in 500 µl whole blood of tumor bearing mice. (B) rKSHV.219 infected cells were detected in murine lymph nodes. Cells from murine lymph nodes were dissociated into single cell suspensions and plated in chamber slides and fixed for fluorescence microscopy for GFP expression. The top panels depict IgG control antibody and native GFP expression, which is quite dim. GFP expression was enhanced with an antibody directed against GFP in the middle and bottom panels. (C) rKSHV.219 infected cells are present in murine spleen. Spleen from a tumor bearing mouse was excised, dissociated with collagenase IV and cultured in puromycin containing selective medium. GFP expressing LANA positive cells grew from the splenic cell culture.

EM visualization of HVLPs prompted us to wonder if rKSHV.219 could be detected in distant non-tumor tissues of the murine host. Although the levels of rKSHV.219 in non-tumor sites were low, viral DNA in the bone marrow and whole blood of tumor bearing mice were detectable (Figure 6A). GFP positive cells were detected in the lymph nodes of tumor bearing mice (Figure 6B), and GFP positive cells were cultured and expanded from the spleen of another tumor bearing mouse (Figure 6C).

**Figure 7:**
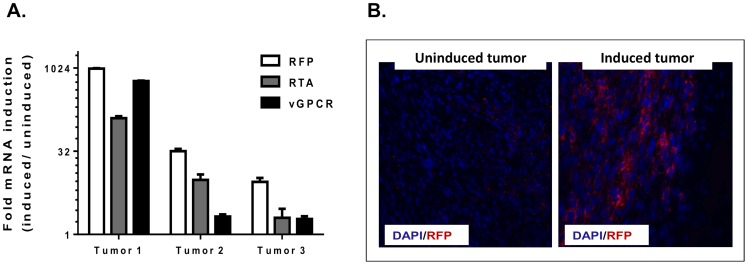
Viral lytic replication in the tumors is enhanced with SAHA, a clinically approved HDAC inhibitor. Three mECK^null^.rK133 tumor bearing mice were treated with 50 mg/kg/day of SAHA for 4 days via intraperitoneal injection. Another set of mice were injected with an equal volume of DMSO, the vehicle. (A) RNA was isolated for qRT-PCR studies of viral lytic gene expression. Three induced tumors were compared to one uninduced tumor. *In vivo* viral lytic gene expression, including RFP, was increased in mice treated with SAHA relative to the untreated tumor. Error bars represent the experimental SD of duplicate wells. (B) DMSO treated and SAHA treated tumors were snap frozen in OCT for frozen sections. Immunoflourescence for RFP was performed to confirm the increase in transcript correlated with an increase in protein expression. RFP was enhanced in tumors undergoing viral lytic replication as a result of the SAHA treatment. Representative tumors are shown.

An emerging antitumor approach for latently infected herpesviral cancers is to combine drugs that induce lytic replication with compounds that target viral lytic proteins and/or enhance the lethality of lytic replication [35]. To determine the ability of the tumors to be pharmacologically induced into lytic replication, mice were treated with a 4 day course of Vorinostat (SAHA) a FDA approved HDAC inhibitor currently being tested in viral and non-viral lymphomas. Compared to uninduced mice, qRT-PCR studies confirmed increased viral lytic gene expression (Figure 7A). Using an anti-RFP antibody to enhance the detection of lytic rKSHV.219, frozen sections were stained to gauge the extent of lytic induction. We found that in tumors from untreated mice, a minority of cells were undergoing lytic replication as determined by RFP expression (Figure 7B, left panel). However, tumors harvested from SAHA treated mice showed robust expression of RFP throughout the tumor, suggesting that the increase in viral lytic RNA expression was due to a global reactivation of viral lytic replication (Figure 7B, right panel).

### rKSHV.219 infection of primary bone marrow-derived cells results in a population KSHV-infected cells with similar transcriptional profile as the mECK^null^.rK133 and other murine endothelial cell types

**Figure 8:**
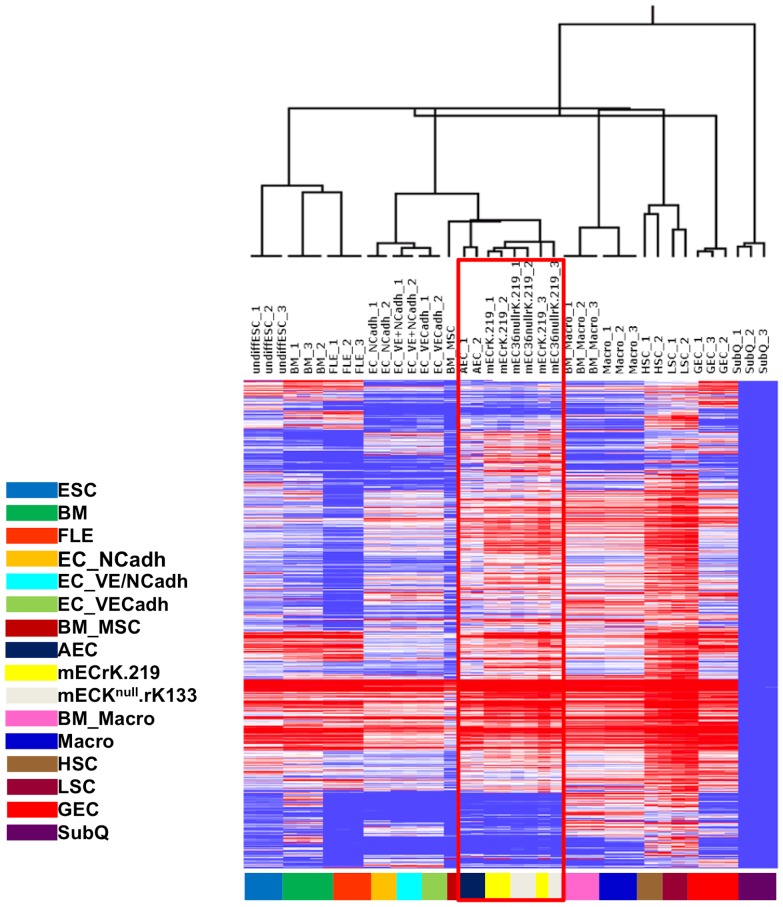
Microarray analysis rKSHV.219-infected murine cells confirms that the primary bone marrow-derived cells and mECK^null^. rK133, are closely related cells of the endothelial lineage. Dendrogram and heatmap of microarray data comparing the host transcriptome of mECK^null^. rK133 cells (denoted as mEC36nullrK.219 in the figure) and mECrK.219 (denoted as mECrK.219 in the figure) to other murine cells of various lineages: ESC, embryonic stem cells; BM, total bone marrow; FLE, fetal liver erythroblasts; EC_NCadh, N-cadherin expressing endothelial cells; EC_VE+NCadh, VE+N-cadherin expressing endothelial cells; EC_VECadh, VE-cadherin expressing endothelial cells; BM_MSC, bone marrow derived mesenchymal stem cells; AEC, aortic endothelial cells; mECrK.219, primary bone marrow-derived mEC infected with rKSHV.219; mECK^null^.rK133, mECK^null^ cells infected with rKSHV.219 and then enriched for Prominin-1; BM_Macro, bone marrow-derived macrophages; Macro, peripheral macrophages; HSC, hematopoietic stem cells; LSC, leukemia stem cells; GEC, glomerular endothelial cells; SubQ, subcutaneous tissue. Similar to the mECK36 cells [9], the mECK^null^.rK133 are of the endothelial lineage and cluster with murine aortic endothelial cells.

To determine if we could recreate the model from primary cells and *de novo* infection with the rKSHV.219 as this approach has the potential to open up new avenues of research for KSHV-pathobiology using genetically modified mice. Bone marrow was flushed from tumor naïve mice, and adherent cells were cultured in endothelial cell growth medium in a similar manner as previously described for the mECK36 cells [28]. After 2 weeks of expansion, primary cells were infected with high titer rKSHV.219, and infected cells were puromycin selected. The result was a population of cells that were >98% GFP positive and expressed LANA in the classic nuclear punctate pattern (data not shown). We termed these cells, mECrK.219. To gain a better understanding of the similitudes between the original mECK36 and the newly created mECrK.219 and mECK^null^.rK133 cells, we interrogated the transcriptional profiles using multiple murine cell lineages for comparison, including: undifferentiated embryonic stem cells, hematopoietic stem cells, whole bone marrow, differentiated endothelial cells expressing N-cadherin and/or E-cadherin, bone marrow-derived and peripheral macrophages, glomerular endothelial cells and aortic endothelial cells. We found that mECK^null^.rK133 cells clustered most closely to mature aortic endothelial cells, but also to differentiated VE-cadherin expressing endothelial cells and bone marrow-derived mesenchymal stem cells (Figure 8), suggesting that they belong to the endothelial cell lineage.

### Primary murine marrow resident cells infected with rKSHV.219 form KS-like tumors in immunocompromised mice

**Figure 9:**
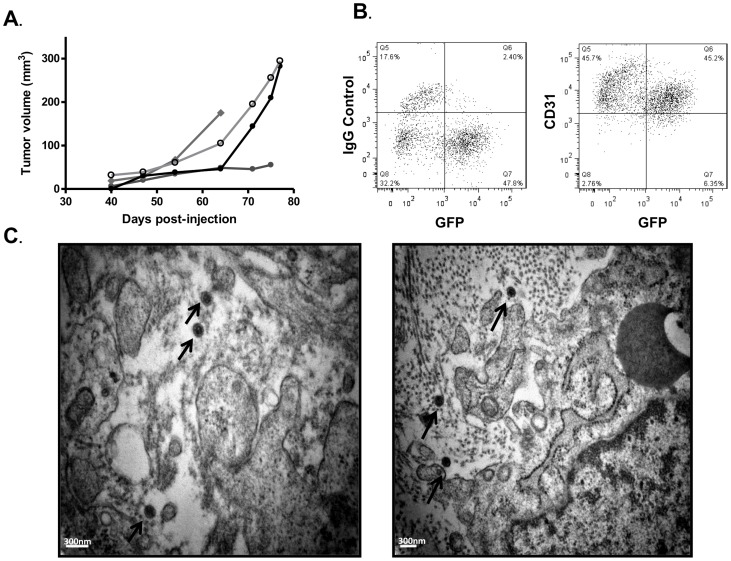
Newly isolated murine bone marrow-derived cells infected with rKSHV.219 generate productively infected tumors in nude mice. (A) Graph depicting growth kinetics in athymic nu/nu mice. 3×10^6^ mECrK cells were subcutaneously injected into the hind flanks of 4 athymic nu/nu mice. Within 4–6 weeks solid tumors were palpable and growth was monitored by caliper measurements. (B) mECrK tumor cells express CD31. Cells from a dissociated tumor were immunostained for CD31, an angiogenic marker and analyzed by flow cytometry for GFP/CD31 co-expression. About half of the CD31+ cells are also rKSHV.219 infected as determined by GFP expression. The vast majority of rKSHV.219 infected cells also expressed CD31. (C) Tumors were excised and fixed in gluteraldehyde for TEM imaging. TEM revealed the presence of herpesvirus-like particles *in vivo*. Bar = 300 nm.

To determine if the mECrK.219 cells were tumorigenic, 3×10^6^ cells were subcutaneously injected into the flanks of immunocompromised mice. Approximately 14 weeks later, palpable GFP expressing, KSHV-infected, KS-like tumors began to grow with similar kinetics to the original mECK^null^.rK cells prior to CD133 enrichment (Figure 9A). Further, similar to the mECK^null^.rK133 tumors, mECrK.219 tumor cells were spindle-shaped *in vivo* (data not shown), approximately 50% of the tumor cells expressed GFP (Figure 9B), indicating KSHV infection, and the majority of those expressed the pan endothelial marker, CD31/PECAM, on the cell surface (Figure 9B). Excised mECrK.219 tumors were analyzed by TEM and found to contain HVLPs of the same size and morphology as was seen in the mECK^null^.rK133 tumors (Figure 9C). Collectively, our results indicate that our method of cell isolation and culture are suitable methods for the generation of productively infected KS-like tumors in immunocompromised mice.

## Discussion

Herein, we described the generation of two murine model of Kaposi's sarcoma able to overcome the main limitations of our previous model derived from murine bone marrow-derived endothelial cells (mEC) transfected with BAC36 (mECK36). For the first model, we replaced the BAC36 with an infectious recombinant KSHV. For the second, we *de novo*-infected primary bone marrow derived murine cells. In both cases we obtained populations displaying KS-like tumors that are productive of viral particles phenotypically similar to KSHV; they expressed genes belonging to the lytic program, which could be further enhanced by HDAC inhibitors and spontaneously manifested the presence of herpesvirus-like particles upon EM analysis.

Although BAC36 transfection into mEC resulted in a cell population capable of forming KS-like lesions in immunocompromised mice, accompanied by increased lytic gene expression, we were able to neither identify nor recover BAC36 generated virus particles in cell culture, tumor sections or explanted materials [9]. This inherently limited the ability of the model to be used for therapeutic testing of targeted antiviral agents as the ability to target viral replication and virion formation, could not be assessed. For example, we recently used virion formation as one of our measures of antiviral activity when we showed that the combination of HDAC and proteasome inhibitors had potent antitumor and antiviral effects in a KSHV-infected primary effusion lymphoma (PEL) NOD/SCID mouse model [35]. Thus, to be able to test this and other antiviral strategies devised in our lab [35], [47], in a KS model, we desired a model capable of virus production as one way of assessing outcomes of KSHV replication.

The inability of the BAC36 system to lead to productively infected tumors *in vivo* could have several explanations. Regarding the BAC36 system, there are both reports showing the ability to recover BAC36 virions from human cells [48], [49] as well as reports depicting genetic modifications of BAC36 that could compromise its ability to faithfully reproduce KSHV lytic replicative abilities [48], [50]. Regarding the use of murine cells, there have been reports of productive infections in the lymphocyte lineage *in vivo* and reports showing the inability of KSHV to carry out productive murine infections *in vitro* [51], [52]. Yet none of these reports addressed the ability of the virus to replicated in a *in vivo* murine endothelial-like environment which we showed was biologically unique from the standpoint of leading to oncogenic consequences [39]. Thus, we hypothesized that by replacing the BAC36 construct in our mECK36 cells with rKSHV.219, a replication competent recombinant KSHV [21], we may address the question of whether the reason for the abortive lytic phenotype was our BAC36 construct or the host. Replacing the BAC36 with rKSHV.219 was accomplished by culturing the mECK36 cells without hygromycin selection for about four months, thus allowing for the loss of the episomal BAC36, and creating the mECK^null^ cells. While it is possible that there were host genetic changes that occurred during this process, the cells remained contact inhibited in culture, did not form colonies in soft agar, and most importantly, they no longer formed tumors when subcutaneously injected into nude mice for up to 3 months, indicating that any changes that may have occurred during the process were not significant to affect the phenotype in a way that it will interfere with detecting KSHV-dependent tumorigenesis.

In fact, we found that rKSHV.219 restored tumorigenicity of the mECK^null^ cells but tumor growth from the mECK^null^.rK was too variable to yield a useful, more reliable animal model. Prominin-1/CD133 is a marker of EPCs [44], [53], [54], putative KS spindle-cell precursors [13], [14], [22], and has been shown by Liu *et al* to be upregulated upon KSHV infection of lymphatic endothelial cells [9], [45]. Our results displaying variable tumor formation from the mECK^null^.rK cells suggested the possibility of the existence of a limited number of true oncogenic cells. Therefore, we chose to enrich for the EPC marker CD133/Prominin-1. Interestingly, these expanded CD133-enriched cells, resulted in mECK^null^.rK133 cells that exhibited a seven-fold enrichment even after 30 days in culture. Most importantly, the mECK^null^.rK133 cells were reproducibly and predictably capable of forming KS-like tumors.

It has been shown that KSHV infection of blood vascular endothelial cells induces lymphatic vascular endothelial reprogramming, leading many to suggest that KSHV-induced reprogramming contributes to the heterogeneity of markers on KS spindle cells [9], [55], [56]. In our model, KS-associated angiogenic endothelial heterogeneity was noticed as mECK^null^.rK133 tumors were found to express markers implicated in KS pathogenesis such as the vascular marker, VEGF-R2, and podoplanin, a lymphatic endothelial marker [57], [58]. These endothelial-lineage spindle-like cells harbored the viral DNA as an episome and expressed LANA in a classic punctate nuclear pattern. Importantly, the virus expressed transcripts that spanned the entire replicative cycle and viral genome, likely underpinning the production of herpesviral like particles (HVLPs). Considering the extensive testing of mEC for numerous murine viruses (see Materials and Methods), the HVLPs are strongly suggestive of KSHV virions. We found that both intracellular and extracellular HVLPs strongly resemble KSHV at varying stages of maturation, including empty capsids, virus-like particles inside intracellular vesicles, some budding from cellular membranes and more mature particles with glycoprotein spikes [49], [59]. EM visualization of rKSHV-like virions prompted us to wonder if rKSHV.219 could be detected in distant non-tumor tissues of the murine host. Various methods to detect presence of virus were used including: viral DNA quantification, RNA detection, and cellular LANA and GFP protein expression. Using these methods, we found evidence of the virus in whole blood, bone marrow, spleen and lymph nodes. Whether these cells represent migration of rKSHV.219-infected cells from the primary tumor, or *de novo* infection *in vivo*, remains to be determined. Collectively, these data suggest that mECK^null^.rK133 tumors recapitulate important viral and host aspects of KS pathobiology.

Although KSHV has the potential to express numerous viral proteins that could be targeted for therapeutic purposes and productive replication is generally considered cytopathic, the primarily latent nature of KSHV leaves few viral targets of therapy. Therefore, an emerging antiviral strategy involves combining drugs that induce lytic replication with compounds that target viral lytic proteins and/or enhance the lethality of lytic replication [35], [60], [61]. This is exemplified by our recent study showing strong antitumor response with SAHA and Btz, where both compounds functioned as lytic inducers of KSHV replication, while Btz also functioned as a potent antiviral by halting the full lytic cycle. Importantly, we found that in our new moue model of KS, SAHA strongly enhanced viral lytic replication *in vivo* as determined by increased RFP expression and lytic gene transcription relative to control treated mice [35].

We found that mECK^null^.rK133 cells still provided a reliable system in which to study KSHV biology and KS therapeutic candidates, as mECK^null^ were not tumorigenic after episome loss, indicating that mECK^null^.rK133 tumorigenicity remained strictly KSHV-dependent. However, sought to determine if we could recreate a murine KSHV tumorigenesis model from primary cells and *de novo* infection with the rKSHV.219. The significance of this approach is that it has the potential to open up new avenues of research for KSHV pathobiology. Reproducing the model from primary bone marrow-isolated cells under conditions of *de novo* infection would allow for the potential to use genetically engineered mice with either knock-in or knock-out phenotypes, allowing for more robust experimentation regarding contribution of host genes in the pathogenesis of KS.

Since *de novo* infected mECrK.219 and mECK^null^.rK133 were obtained by disparate methods, separated by a significant amount of times, we resorted to genomic analysis to gauge the similarities between the mECK^null^.rK133 cells, which were already shown to have characteristics similar to KS-spindle cells, and the newly created mECrK.219. We interrogated the transcriptional profiles using multiple murine cell lineages for comparison, including: undifferentiated embryonic stem cells, hematopoietic stem cells, whole bone marrow, differentiated endothelial cells expressing N-cadherin and/or E-cadherin, bone marrow-derived and peripheral macrophages, glomerular endothelial cells and aortic endothelial cells. We found that mECK^null^.rK133 and the newly derived mECrK.219 cells clustered most closely to mature aortic endothelial cells, but also to differentiated VE-cadherin expressing endothelial cells and bone marrow-derived mesenchymal stem cells. Remarkably, both of the cell populations, the mECK36, which was derived many years ago (transfected with the BAC36, with subsequent replacement of BAC36 by rKSHV.219 infection), and the mECrK.219, derived recently and infected with the rKSHV.219, cluster so closely together as to be almost indistinguishable from each other by microarray.

We found that both the mECK^null^.rK133 and the mECrK.219 formed vibrantly green subcutaneous KS-like tumors that produced HVLPs *in vivo*, indicating that the cells are able to support full lytic replication *in vivo*. However, as with the mECK^null^.rK133, we were unable to detect or recover rKSHV.219 from lytically induced cultures of mECrK.219, suggesting that the host environment is necessary for complete viral lytic replication to occur. With these cellular isolation procedures, culture and infection methods, we now can move forward with isolating and infecting bone marrow-derived cells from mice that have been genetically modified in multiple aspects important to KSHV pathobiology, such as angiogenesis.

It was remarkable that cells isolated and infected at such disparate times and with such different methods, one being manipulated by BAC36 transfection, with subsequent loss of the BAC36 and then re-infected with rKSHV.219, and the other being primary isolated and rKSHV.219 infected could give express such similar transcriptional profiles and generate phenotypically similar KS-like tumors *in vivo*. Two plausible explanations could account for this outcome: (1) both rKSHV.219 infection and BAC36 transfection were able to establish an infection in, and promote survival of, spindle cell-like progenitor cell(s) isolated from the bone marrow or, (2) KSHV infection induced transdifferentiation of the spindle-like progenitor cell(s) to the same oncogenic endothelial cell lineage. Importantly, both explanations point to the presence of a KS-like spindle cell progenitor among the bone marrow population that was targeted both by BAC36 transfection and rKSHV.219 infection. These results are also consistent with studies showing the oncogenic consequence of KSHV infection of rat bone marrow mesenchymal cells [40].

In summary, we have constructed two productively infected murine models of KS; one from a mouse line established previously in our lab and another from primary isolated bone marrow derived cells. By reproducing the tumorigenic phenotype in *de novo* infected primary marrow-derived murine cells, we have identified a method that sets a foundation for the systematic identification of the KS progenitor cell. Further, our isolation and culture system of primary bone marrow cells and rKSHV.219 infection opens the possibility of delineating KSHV biology and KS pathogenesis with mice genetically engineered to aberrantly express genes important to KS pathophysiology, such as those involved in angiogenesis and inflammation. Finally, these types of productively infected, lytically inducible models should open avenues of research that include *in vivo* basic science studies of KSHV reactivation and replication and should be excellent systems in which to test novel KSHV-targeted antitumor strategies.

## Materials and Methods

All animal work was conducted according to relevant national and international guidelines and conducted under the University of Miami Miller School of Medicine's Institutional Animal Care and Use Committee (IACUC) approved protocols in AAALAC certified facilities.

### Chemical reagents

Vorinostat was obtained from LC Laboratories. Trichostatin A, puromycin, and DMSO were obtained from Sigma-Aldrich. All reagents were sterile filtered prior to use.

### Generation and maintenance of mECK^null^.rK133 and mECrK.219

mECK^null^.rK133 were generated by allowing mECK36 cells to lose the BAC36 construct through serial passage in culture without hygromycin selection. This resulted in mECKSHV^null^ (mECK^null^) cells. mECK^null^ were then infected with rKSHV.219 at an MOI of 1 and expanded under puromycin selection at 1–2ug/mL, these are mECK^null^.rK cells. Finally, Prominin-1 (CD133) expressing cells were enriched from the mECK^null^.rK cells using magnetic beads (Multenyi Biotec) as per manufacturer's protocol and expanded under puromycin selection at 1-2ug/mL. These are termed mECK^null^.rK133 cells. Primary cells were isolated from the bone marrow of 10 week old *athymic* NCr-nu/nu mice from the National Cancer Institute (National Institutes of Health). Mouse femurs were flushed with PBS, bone marrow cells were put in culture in for 3 days. Non-adherent cells were washed away and medium highly enriched for endothelial growth factors was replaced every 3 days for two weeks to allow for expansion of the adherent cells. The cells were then infected with rKSHV.219 in the presence of polybrene (8ug/ml) for 2 hours. 2 days later, puromycin was added to the culture to select and expand the infected cells. All murine cells were cultured in endothelial cell (EC) growth medium (mEC medium): DMEM supplemented with 30% FBS (Gemini Bioproducts), 0.2 mg/ml Endothelial Cell Growth Factor (ECGF) (Sigma-Aldrich), 0.2 mg/ml Endothelial Cell Growth Supplement (ECGS) (Sigma-Aldrich), 1.2 mg/ml heparin (Sigma-Aldrich), insulin/transferrin/selenium (Invitrogen), 1% penicillin-streptomycin (Invitrogen), and BME vitamin (VWR Scientific). NIH3T3 cells were purchased from American Type Culture Collection. HEK293 cells were cultured in DMEM with 10% FBS (Gemini Bioproducts) and 1% penicillin-streptomycin (Invitrogen). All cell lines were PCR tested for: Mycoplasma spp., Sendai virus, mouse hepatitis virus, pneumonia virus of mice, minute virus of mice, mouse parvovirus (MPV1, MPV2, MPV3), Theiler's murine encephalomyelitis virus, murine norovirus, reovirus 3, mouse rotavirus, ectromelia virus, lymphocytic choriomeningitis virus, polyoma virus, lactate dehydrogenase-elevating virus, mouse adenovirus (MAD1, MAD2), mouse cytomegalovirus.

### Soft agar assay

Base agar was made by combining melted 1% agar with 2× mEC medium to give a 0.5% Agar/1X mEC medium solution. 1.5 mL was added to each well of a 6 well plate and allowed to set. Five thousand cells were plated on top of base agar in 0.7% agar/2X mEC medium in triplicate in 6-well plates. The cells were fed every 3 days with 1 mL of mEC medium (described above). Colonies were photographed at 4 weeks. Only colonies larger than the mean size of the background colonies in the NIH3T3 negative control wells were considered.

### Histopathology and immunofluorescence

Tissue slices of rKSHV.219 infected mouse tumors were fixed overnight in 10% buffered formalin, embedded in paraffin, cut into 5 µm sections, and H&E stained. Frozen sections were fixed in 10% buffered formalin for 10 min. room temperature. They were then washed with PBS and permeabilized with 0.2% Triton-X in PBS for 30 min. at 4°C, washed in PBS, and blocked with 10% goat serum (Dako) for 30 min. Primary antibodies were diluted to 1∶100 in 2% goat serum and incubated at room temperature for one hour. Sections were then washed with PBS and secondary goat anti-rabbit or goat anti-mouse, as appropriate, antibodies conjugated to Cy3 or Cy5 (Molecular Probes, Invitrogen) were added at a 1∶500 dilution for one hour at room temperature. TSA induced cells were used as positive control for RFP antibody specificity. The anti-RFP antibody was counterstained with a Cy5 labeled secondary antibody. RFP visualized with the Cy3 channel co-localized with Cy5, indicating that the RFP antibody specifically stained RFP expressing cells. Uninduced cells, those expressing only GFP, were not stained by the anti-RFP antibody. Sections were washed, allowed to dry and mounted with ProLong Gold with DAPI (Invitrogen). All relevant isotype controls were included for negative controls. LANA antibody was purchased from Abcam. Images were taken using Zeiss Axiovision 4.8.2 with a Hamamatsu ORCA-R2 CCD camera and Zeiss Axiovert 200 M inverted fluorescence microscope.

### Transmission electron microscopy

Tumors were excised and fixed overnight in 2.5% glutaraldehyde, 100 mM sucrose, 0.05 M phosphate (PO_4_) buffer. After fixation, the segments were rinsed in 3 changes of 0.15 M PO_4_ buffer, pH 7.2 for 10 min. each. Samples were then fixed with 1% osmium tetroxide in 0.1 M PO_4_ overnight at 4°C. Following fixation, the segments were rinsed 3 times in 0.15 M PO_4_ buffer for 10 min. 3 times each. The tumors were then dehydrated through a graded ethanol series and rinsed twice in propylene oxide for 5 min. each. A 1∶1 mixture of propylene oxide: Epon Araldite with DMP-30 (E/A) was added overnight at room temperature. Fresh E/A was added and the tissue was desiccated for 5 hours. Tissue was moved to embedding molds in fresh E/A, uncovered in 64°C oven overnight before embedding, sectioning and mounting on copper grids. The sections were viewed with a Philips CM-10 Transmission Electron microscope.

### Microarray analysis

Using the Affymetrix MoGeneST_1.0 array, we compared gene expression levels of the mECK^null^.rK133 cells and mECrK cells to data from 40 microarray samples representing 16 different mouse cell and/or tissue types also run on MoGeneST_1.0 array collected from GEO datasets. GEO accession numbers are as follows: Aortic Endothelial cells (AEC: GSM686077, GSM686078), Bone marrow derived mesenchymal stem cells (BM_MSC: GSM757750), N-Cadherin expressing endothelial cells (EC_NCadh: GSM859433, GSM859434), VE-Cadherin expressing endothelial cells (EC_VECadh: GSM859435, GSM859436), VE+N-Cadherin expressing endothelial cells (EC_VE+NCadh: GSM859437, GSM859438), total bone marrow (BM: GSM821069, GSM821070, GSM821071), undifferentiated embryonic stem cells (undiffESC: GSM839393, GSM839394, GSM839395), fetal liver erythroblasts (FLE: GSM842148, GSM842149, GSM842150), bone marrow-derived macrophages (BM_Macro: GSM787745, GSM787746, GSM787747), peripheral macrophages (Macro: GSM466436, GSM466437, GSM466438), hematopoietic stem cells (HSC: GSM862182, GSM862184), leukemia stem cells (LSC: GSM862186, GSM862188), glomerular endothelial cells (GEC: GSM532931, GSM532932, GSM532933) and subcutaneous tissue (SubQ: GSM905121, GSM905122, GSM905123). Each sample was processed through QA/QC on GeneSpring 12 software (Agilent technologies). A batch bias was noted since the data was derived by many groups. Each batch was individually Quantile normalized and log2 transformed to median of all samples within the batch. The combined data was corrected for batch bias using the using the ComBat [62] module on R/Bioconductor package. In order to clearly demarcate the clusters of samples, 7549 probes with most variation (SD≥1.5) among all the 40 samples were subset. A hierarchical clustering was performed on the subset using GeneSpring. For clustering the Pearson centered similarity measure method with a centroid linkage rule was used.

### RNA Isolation and quantitative real-time RT-PCR (qRT-PCR)

Total RNA was isolated using the RNeasy Plus kit or the AllPrep RNA, DNA, protein kit (Qiagen) for concomitant RNA/DNA isolation studies. DNase treatment of eluted RNA was done with Turbo DNase (Ambion) as per manufacturer's instructions. 500 ng of RNA was transcribed into cDNA using random primers and the Reverse Transcription System (Promega) according to the manufacturer's instructions. qRT-PCR reactions were run using SYBR Green PCR Master Mix (VWR) on an ABI Prism 7000 Sequence Detection System (Applied Biosystems). Dissociation curve analysis verified specificity of products. cDNA products of selected KSHV genes were run on a 3% agarose gel which also confirmed the dissociation curve indicating that primers were specific for single amplicons. Reverse transcriptase negative and non-template controls were run to verify purity of sample. Data were analyzed using the ΔΔCt method where target gene expression is normalized to the housekeeping gene by taking the difference between Ct values for target gene and housekeeping gene (ΔCt). This value was then compared to that of the normalized control sample (ΔΔCt). The fold change was then determined by the formula: 2^-ΔΔCt^. The following primer sets were used:

RFP (5′-AGGACGGCTGCTTCATCTAC-3′, 5′-TGGTCTTCTTCTGCATCACG-3′);

LANA (5′-CCTGGAAGTCCCACAGTGTT-3′, 5′-AGACACAGGATGGGATGGAG-3′);

vCyclin (5′-GCCGCGCTTTTTAACTTCTGAC-3′,5′-AAATAGGCGTGAGGCTTCTGAG-3′);

vFLIP (5′- GGATGCCCTAATGTCAATGC -3′,5′- GGCGATAGTGTTGGGAGTGT -3′);

Kaposin (5′-AGGCTTAACGGTGTTTGTGG -3′, 5′-GTTGCAACTCGTGTCCTGAA -3′);

RTA (5′-CAAGGTGTGCCGTGTAGAGA-3′, 5′-TCCCAAAGAGGTACCAGGTG-3′);

ORF45 (5′-CATGGGATGGGTTAGTCAGG -3′, 5′-GGGTCGCTGTATGGTGAACT -3′);

ORF21 (5′-ACGCCGTGTGCGGGATCTTG -3′, 5′-GACGCACCAAGTGAGTGCCCC -3′);

ORF36 (5′-CCCCCGGTGTGCCCTGAAAC -3′, 5′-ATCCTGGTGCGTGCACTGCC -3′);

vCCL2/K4 (5′-CGTTTTATGCTGCGTTAG-3′,5′-AGTTTTTGGAAGGGTCTGC-3′);

vIRF1 (5′-GGAAGAACAATGCGTGGAATG-3′, 5′-CGACTGGCTTGTCGTCAGTA-3′);

vIL6 (5′-TGCTGGTTCAAGTTGTGGTC-3′, 5′-ATGCCGGTACGGTAACAGAG-3′);

ORF55 (5′-GCATTCCCCGGCCCTTTTGTTTA-3′,5′-CTCGCGGCGGTATGTCGTCTCC-3′);

ORF49 (5′-CGAGAAGGCCCCTTAAAGAT-3′, 5′-GGTACGTGGCAGTCTGGATT-3′);

ORF57 (5′-GGGTGGTTTGATGAGAAGGA-3′, 5′-CGCTACCAAATATGCCACCT-3′);

ORF74 (5′-TGTGTGGTGAGGAGGACAAA-3′, 5′-GTTACTGCCAGACCCACGTT-3′);

K7/vIAP (5′-CTGCCGCTTCACCTATGGATTTT-3′, 5′-AACTGGCCTGGAGATTGAA-3′);

K8.1 (5′-CACCACAGAACTGACCGATG-3′, 5′-TGGCACACGGTTACTAGCAC-3′);

ORF8/gB (5′-CTGGGGACTGTCATCCTGTT-3′, 5′-ATGCTTCCTCACCAGGTTTG-3′);

GAPDH (5′-ACCCAGAAGACTGTGGATGG-3′, 5′-CACATTGGGGGTAGGAACAC-3′);

TBP (5′-CAGCCTTCCACCTTATGCTC-3′, 5′-TTGCTGCTGCTGTCTTTGTT-3′);

GFP (5′-ACGTAAACGGCCACAAGTTC-3′, 5′-AAGTCGTGCTGCTTCATGTG-3′)

### Flow cytometry

Tumors were excised, minced and further dissociated at 37°C with collagenase IV (Worthington Biochemicals) in DMEM, 0.5% bovine serum albumin, 1X pen/strep/fungi, and 2 µL/mL DNAse (Ambion) by shaking at 180 rpm for 30–60 min. Dissociation was complete after final trituration using a 5 mL pipette and then filtering the material through 70 uM and then 40 uM filters. Single cells were stained with anti-CD31 (BD Biosciences) or an isotype control antibody for 30 min. at 4°C. After washing, a secondary APC-conjugated antibody was added for 30 min. at 4°C. Cells were then washed with PBS and fixed with Cytofix fixation buffer (BD Biosciences). For analysis of CD133 expression on cultured cells, cells were diluted in FACS buffer of PBS containing 2% FBS and 1 mM EDTA. They were preincubated with Fc receptor blocker (BD Biosciences) for 20 min at 4°C and then washed thrice. APC-conjugated anti-Prominin-1 antibody or the isotype control (Multenyi Biotec) were diluted in FACS buffer at 1∶200, and added to the cells for 30 min incubation at 4°C. Cells were then washed thrice in cold PBS and fixed with fixation buffer (BD Biosciences). All flow cytometric analysis was performed on a Becton-Dickinson LSR analyzer (BD Biosciences) and analyzed using FlowJo (Tree Star, Inc.) software.

### Tumorigenesis studies

3×10^6^ cells were injected into the hind flank of immunocompromised mice. Mice were monitored daily. For growth kinetics, tumors were measured by caliper and volume was calculated using the formula: l×w^2^×0.52. For *in vivo* lytic induction, groups of 3 mice with tumor volumes of 50–150 mm^3^ were treated with intraperitoneal injections for 4 days with 50 mg/kg SAHA. On day 5, mice were sacrificed using carbon dioxide euthanasia provided by certified equipment in our animal care facilities. Tumors were snap frozen in LN_2_ for RNA extraction or in OCT for frozen section preparation. All animal work was conducted according to relevant national and international guidelines and conducted under the University of Miami Miller School of Medicine's Institutional Animal Care and Use Committee (IACUC) approved protocols in AAALAC certified facilities

### Statistical analysis

All experiments were performed at least in triplicate unless mentioned otherwise. Numerical data are expressed as mean ± SD. Two-sided Student's *t*-test was used to analyze two group comparisons. When P values were calculated, a p<0.05 was considered significant.
